# Rapid detection of *Plasmodium falciparum* with isothermal recombinase polymerase amplification and lateral flow analysis

**DOI:** 10.1186/1475-2875-13-99

**Published:** 2014-03-15

**Authors:** Sebastian Kersting, Valentina Rausch, Frank Fabian Bier, Markus von Nickisch-Rosenegk

**Affiliations:** 1 Department of Nanobiotechnology and Nanomedicine, Fraunhofer Institute for Biomedical Engineering (IBMT), Branch Potsdam, Am Muehlenberg 13, 14476 Potsdam-Golm, Germany; 2 Institute of Biochemistry and Biology, University of Potsdam, Karl-Liebknecht Strasse 24-25, 14476 Potsdam-Golm, Germany

**Keywords:** *Plasmodium falciparum*, Recombinase polymerase amplification, RPA, PCR, Lateral flow, Point-of-care testing, Rapid test, Isothermal nucleic acid amplification

## Abstract

**Background:**

Nucleic acid amplification is the most sensitive and specific method to detect *Plasmodium falciparum*. However the polymerase chain reaction remains laboratory-based and has to be conducted by trained personnel. Furthermore, the power dependency for the thermocycling process and the costly equipment necessary for the read-out are difficult to cover in resource-limited settings. This study aims to develop and evaluate a combination of isothermal nucleic acid amplification and simple lateral flow dipstick detection of the malaria parasite for point-of-care testing.

**Methods:**

A specific fragment of the 18S rRNA gene of *P. falciparum* was amplified in 10 min at a constant 38°C using the isothermal recombinase polymerase amplification (RPA) method. With a unique probe system added to the reaction solution, the amplification product can be visualized on a simple lateral flow strip without further labelling. The combination of these methods was tested for sensitivity and specificity with various *Plasmodium* and other protozoa/bacterial strains, as well as with human DNA. Additional investigations were conducted to analyse the temperature optimum, reaction speed and robustness of this assay.

**Results:**

The lateral flow RPA (LF-RPA) assay exhibited a high sensitivity and specificity. Experiments confirmed a detection limit as low as 100 fg of genomic *P. falciparum* DNA, corresponding to a sensitivity of approximately four parasites per reaction. All investigated *P. falciparum* strains (n = 77) were positively tested while all of the total 11 non-*Plasmodium* samples, showed a negative test result. The enzymatic reaction can be conducted under a broad range of conditions from 30-45°C with high inhibitory concentration of known PCR inhibitors. A time to result of 15 min from start of the reaction to read-out was determined.

**Conclusions:**

Combining the isothermal RPA and the lateral flow detection is an approach to improve molecular diagnostic for *P. falciparum* in resource-limited settings. The system requires none or only little instrumentation for the nucleic acid amplification reaction and the read-out is possible with the naked eye. Showing the same sensitivity and specificity as comparable diagnostic methods but simultaneously increasing reaction speed and dramatically reducing assay requirements, the method has potential to become a true point-of-care test for the malaria parasite.

## Background

Malaria causes the most deaths worldwide of all parasitic diseases, resulting in an estimated 660,000 casualties annually. *Plasmodium falciparum* is with 90% of all cases the main source for infection in humans of all *Plasmodium* species [1]. Effective treatment and surveillance of malaria is only possible with a reliable and sensitive diagnostic set-up. At present, the parasite is routinely detected by microscope techniques that require instrumentation and a trained eye. Also rapid diagnostic tests, usually based on antigen detection, for point-of-care testing are available. However these tests, like microscopy, often lack in sensitivity or clarity of results. Molecular techniques such as polymerase chain reaction (PCR), which amplify a specific nucleic acid sequence, have been developed over the past years. These methods have the advantage that they can detect low level of parasitaemia [2] or give additional information on the infection in a single assay [3,4]. Nevertheless, the PCR still remains laboratory based due to the high complexity of the assay and the still not-far-enough advanced portable devices for point-of-care testing. Furthermore, the continuous electricity dependency for the thermocycling process and the rather expensive equipment necessary for the PCR machinery makes it difficult to conduct this method in resource-limited settings, which might occur in some areas especially in developing countries and thus is restricted to be a confirmatory technique in laboratory diagnostics [5].

Lately several isothermal nucleic acid amplification techniques have been established which do not require the thermocycling process but run on a constant temperature. Examples are the loop-mediated isothermal amplification (LAMP) for the detection of *P. falciparum*[6], *Plasmodium vivax*[7] or for four species of human malaria parasites [8] and the real-time quantitative nucleic acid sequence based amplification (real-time QT-NASBA) for the quantification of rRNA samples of three different *Plasmodium* strains [9]. These methods drastically simplified the assay set-up and improved the speed for nucleic acid amplification in comparison to a standard PCR.

**Figure 1:**
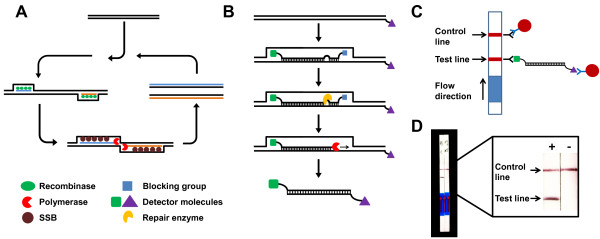
**Recombinase polymerase amplification and lateral flow detection.** Reaction principle of the recombinase polymerase amplification (RPA). The two oligonucleotide primers form a complex with the recombinase proteins (green). This complex is able invade the target DNA and directs the primer to homologous sequences. A continuous amplification at 38°C takes place by strand-displacement synthesis catalyzed by a DNA polymerase (red) while single-strand binding proteins (SSB) (brown) stabilize the displaced strand **(A)**. During LF-RPA the reverse primer carries an antigenic tag at the 5′end and a modified probe is added in the reaction. The modified probe is labelled with a different antigenic tag at the 5′end and a 3′end polymerase blocking group. Additionally the probe contains an abasic site 30 nt downstream the 5′end. Only when the probe fully binds to a homologous sequence the nuclease (yellow) is able to cut through the DNA double strand and release the blocking group. Thereby additional polymerase extension substrate is generated and the continuation of strand synthesis results in a dual-labelled amplicon. Amplification and labelling reaction run in the same tube **(B)**. Detection of the labelled RPA amplicon on the lateral flow dipstick by binding to tag-specific antibodies on the test strip and to tag-specific antibodies on gold nanoparticles present in the sample application area. A reddish band is generated on the test line in a sandwich assay manner if the amplicon was successfully generated. Not captured gold particles flow over and are fixed by species-specific antibodies on the control line **(C)**. LF-RPA for *P. falciparum* using 1 ng of genomic DNA and 25 min reaction time. A test band visible to the naked eye is formed and confirms the presence of the RPA amplicon. The control line determines the validity of the test run. Detail enhanced for better view **(D)**.

A novel approach of isothermal amplification could be demonstrated with the recombinase polymerase amplification (RPA) [10]. This method uses two target specific oligonucleotide primers, which are able to bind to the template DNA with the assistance of a recombinase in combination with strand-displacement DNA synthesis (Figure 1A). At temperatures just above room temperature an amplification of complex DNA targets can be achieved in less than 30 minutes. Recently, several applications of this method have been demonstrated for the detection of DNA and RNA targets [10-14] and the integration of the RPA in different instrumentations was shown [15-18].

The analysis of the amplification products usually involves labour-intensive and costly read-out arrangement, for example, electrophoresis systems or sensors and optics for fluorescence detection. For resource-limited settings, simpler systems like lateral flow immunoassays strips are an option. With nucleic acid lateral flow immunoassays (NALFIA), it is possible to detect double-stranded amplicons, which were labelled with two different antigenic tags during the enzymatic reaction [19] (Figure 1B). The recognition is achieved by binding to a tag-specific antibody on the surface at the detection line of the lateral flow strip and by cross-linking it with a second tag-specific antibody on colloid gold nanoparticles, resulting in a coloured signal that can be semi-quantitatively analysed with the naked eye (Figure 1C). For the detection of the RPA amplification product a probe-based system was used [10,17]. The development and evaluation of a combination of isothermal RPA and lateral flow strip detection for *P. falciparum* is described. The presented test reduces the need for instrumentation and complexity of nucleic acid amplification and generates a potential improvement for the point-of-care diagnostics of malaria.

## Methods

### Samples

Genomic DNA of in total 77 *P. falciparum* strains were obtained through the Malaria Research and Reference Reagent Resource Center (MR4) as part of the BEI Resources Repository. Quantity and quality of the extracted DNA was determined by measuring A260 and the ratio of A260/A280 on a Nanodrop ND-1000 spectrophotometer (ThermoScientific, USA). The DNA of 3D7 was adjusted to a working concentration of 1 ng/μl in TE-buffer. Other DNAs of prokaryotic and eukaryotic samples were extracted using the GeneJET Genomic DNA Purification Kit (ThermoScientific, USA) and also tested for quantity and quality.

### RPA reactions in solution

For optimization and primer testing the RPA reactions were first conducted in solution. A typical 50 μl reaction was performed with the TwistAmp Basic kit (TwistDx, UK) and contained 480 nM RPA primers, 1 M betaine and 1x rehydration buffer. To start the reaction 14 mM magnesium acetate was added. Samples were incubated for a typical 25 min at 38°C, if not indicated otherwise, in a Thriller thermoshaker-incubater (PEQLAB Biotechnologie GMBH, Germany) under constant shaking at 300 rpm. Subsequent analysis on 2% agarose-gel (Agarose Broad Range, Germany) stained with ethidium bromide was conducted after purification of the samples with the QIAquick PCR Purification Kit (Qiagen, Germany). Primers were designed using parameters according to the TwistDx instruction manual [20] and Primer-BLAST available at http://www.ncbi.nlm.nih.gov/tools/primer-blast/ combining Primer3 and BLAST global alignment. All oligonucleotides in this study were synthesized by biomers.net (Germany). Various primer combinations and targets were evaluated and one primer pair was selected during the process: PfFwd 5′-GTGTTCATAACAGACGGGTAGTCATGATTGAGT-3′ and PfRev 5′-ACATCTGAATACGAATGCCCCCAAAGATACTCC-3′ resulting in an amplicon with the size of 174 bp targeting a region on the small subunit (SSU) (18S rRNA) gene of *P. falciparum* (GenBank: JQ627152).

### Lateral flow RPA assay

For the adaptation to the lateral flow detection system, a labelling RPA was carried out. Herein, a labelling reverse primer and a lateral flow probe were used. The labelling primer is identical to the PfRev primer but contains a digoxigenin (DIG) at the 5′end. The sequence of the hybridization probe was 5′-Carboxyfluorescein - GTGTTTGAATACTACAGCATGGAATAACAA**X**TATGAATAAGCTAATTATT-Spacer C3-3′ where “X” highlights the insertion of an abasic furan (dSpacer), which was introduced in the probe sequence to mimic an abasic site. A double stranded DNA template is formed when the probe binds to the antisense DNA strand. The nuclease contained in the reaction solution recognizes the abasic site and cleaves the phosphodiester bond and as a consequence releases the blocking group. The polymerase is now able to extend the probe and the amplification process continues. In combination with the unlabelled forward primer, a dual-labelled amplicon is generated which can be analysed directly on the lateral flow dipstick (Figure 1B). The reaction conditions for the single tube lateral flow amplification using the TwistAmp nfo kit (TwistDx, UK) were 420 nM of forward and reverse primer, 120 nM lateral flow probe, 1x rehydration buffer and 1 M betaine. The reaction was adjusted to 50 μl by adding ddH_2_O and used for resuspending the freeze-dried pellet. After adding the template DNA 14 mM magnesium acetate was added to start the reaction. Samples were incubated for 25 min at 38°C in a Thriller thermoshaker-incubater (PEQLAB Biotechnologie GMBH, Erlangen, Germany) under constant shaking at 300 rpm.

### Visualization on lateral flow dipsticks

For the detection of the RPA-generated, labelled amplicon, Hybridetect 2 T (Milenia Biotec GmbH, Germany) dipsticks were used. The reaction product is visualized with gold nanoparticles conjugated with polyclonal anti-carboxyfluorescein antibodies (rabbit) contained on the lateral flow strip at the application pad. The gold nanoparticle/amplicon – conjugate can then bind with the 3′-end to immobilize digoxigenin antibodies on the detection line. A control line with immobilized anti-rabbit antibodies serves as an assay control. Two μl of the RPA reaction were diluted in 98 μl of tris-buffered saline. Dipsticks were directly inserted into this dilution and were incubated at an upright position for 5 min. Pictures were taken in the BioDocAnalyze imagine system (Analytik Jena AG, Germany).

### Evaluation of lateral flow RPA conditions

For sensitivity testing, a dilution of genomic DNA of *P. falciparum* 3D7 to 100 pg, 10 pg, 5 pg, 1 pg, 500 fg, 100 fg and 10 fg per μl was prepared in TE-buffer and a volume of 1 μl was used per reaction. The specificity of the LF-RPA was analysed using excess amounts of gDNA from a panel of eukaryotic and prokaryotic organisms. At least 10 ng of gDNA were used as template in these reactions. In further experiments 50 ng of gDNA of the closely related pathogen *Toxoplasma gondii* and 50 ng of human DNA, which might be present in samples using whole-blood extracts, where utilized for specificity testing. In total 77 strains of *P. falciparum* were investigated using 1 ng of gDNA as template for the amplification. The reaction time was determined by stopping the reaction at 1, 5, 10, 15, 20, 25 and 30 min after the addition of magnesium acetate by immediate dilution and analysis on the lateral flow dipsticks. The reaction conditions for the temperature experiments were according to the above-mentioned lateral flow RPA assay protocol with varying temperatures ranging from 15-50°C. For inhibitory experiments, 1 ng of genomic DNA of *P. falciparum* 3D7 was used in a 50 μl RPA reaction which contained the following inhibitory substances: SDS (0.05% v/v), ethanol (4% v/v), heparin (0.5 U) (Sigma-Aldrich, Germany) and haemoglobin (2 g/l; 20 g/l and 50 g/l) (Sigma-Aldrich, Germany) in which the numbers in brackets indicate the final concentrations in these experiments. Additionally, 10 μl per 50 μl reaction of whole blood (Basis-line VB, Medichem, Germany) and serum (Basis-line S, Medichem, Germany) was utilized for further inhibitory tests.

## Results

### Establishing the lateral flow RPA

In order to establish the RPA, initial experiments were undertaken in single tube reactions without the addition of the probe, to screen primers and test different reaction conditions. Even though the RPA has a PCR-like set-up, PCR primers differ from RPA primers. Longer oligonucleotides are necessary to stimulate and complete priming with support of the recombinase proteins, therefore the designed RPA primers are typically between 30 and 35 nucleotides long. After this initial selection process a second step combined the RPA with lateral flow detection, again with a consideration on signal strength, absence of background and sensitivity. The probes used for lateral flow assays are 46-52 nucleotides long and the abasic site is located 30 nucleotides downstream of the 5′end [20]. For the detection of all *Plasmodium* species several multicopy loci are known. Most PCR methods target the 18S rRNA genes because of the high sequence conservation and the widely available sequence data [2,21]. Further amplification targets with multiple copies include pfr364 [22], *stevor*, *rif*[23] and mitochondrial DNA sequences [24]. These sequences have been demonstrated to be more sensitive and show an even faster amplification due to the higher number of repeats in the genome. Different primers and probes targeting several multicopy loci of *P. falciparum* were screened and as a result a primer pair and probe targeting the 18S rRNA was chosen and used in the follow-up experiments. In Figure 1D the result of a lateral flow RPA (LF-RPA) using 1 ng of *P. falciparum* 3D7 gDNA is depicted. In this example the reaction time was 25 min of amplification at 38°C and an additional 5 min of incubation of the lateral flow dipstick. A clearly visible reddish band at the test line is observed while in the negative control no band is noticeable. The control band on both dipsticks indicates a valid test run. Primer and probes were designed to detect all *Plasmodium* species by their ribosomal RNA genes but for subclassification purposes, another target sequence might be more favourable. Also other multicopy targets might increase sensitivity and reaction speed but with the straightforward set-up of the LF-RPA the method can be adapted to other gene sequences.

### Sensitivity of the LF-RPA assay

**Figure 2:**
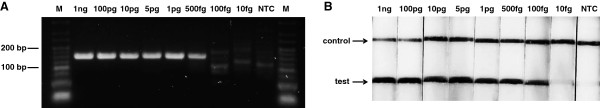
**Reaction sensitivity of the recombinase polymerase amplification.** A serial dilution of genomic DNA from *P. falciparum* 3D7 was used to test the analytical sensitivity of the assay. Positive RPA reaction products (174 bp) can be detect on a stained agarose gel (2%) **(A)**. In the lateral flow format (LF-RPA) the sensitivity was 100 fg of genomic DNA **(B)**. NTC: no template controls contained water.

The detection threshold of the LF-RPA was determined by using a dilution series of *P. falciparum* 3D7 ranging from 1 ng to 10 fg DNA per reaction. Additionally, the RPA reaction was tested for sensitivity without lateral flow probe by subsequent visualization with agarose gel electrophoresis. The method was found highly sensitive with detection limits of 100 fg and 500 fg, respectively, corresponding to approximately four and 20 parasites/reaction (Figure 2). In contrast to LAMP-based amplification techniques with its ladder-like pattern of products, the RPA amplicon can be directly identified by the specific size of the band on the agarose gel (Figure 2A). The instrument-free detection on the lateral flow dipsticks revealed a higher sensitivity than the agarose gel-based detection (Figure 2B). This finding supports previous studies, in which the nucleic acid lateral flow immunoassay in combination with PCR, demonstrates lower or similar detection limits in comparison to gel electrophoresis analysis [25,26]. Moreover the use of probe during the LF-RPA reaction provides an additional starting point for polymerase elongation by functioning as primer upon binding to the target sequence. Especially with low amounts of starting DNA, this might increase the amplification time and sensitivity of the reaction. The reactions performed to determine the sensitivity of the LF-RPA were constantly shaken during the amplification. In particular when working with DNA template concentrations close to the limit of detection, a constant shaking results in more stable signals on the test strips than with the recommended single flicking/shaking event. When the shaking is completely omitted a reduced sensitivity and reaction efficiency of the assay can be observed. For the development of a POCT test this has to be taken into account in order to enable the best performance of the amplification reaction. However, the LF-RPA has a lower limit of detection comparable to nested PCR methods for *P. falciparum* and surpasses the analytical sensitivity of malaria microscopy of thin films (200 parasites/μl) [27] and rapid diagnostic tests (RDTs) (>100 parasites/μl) [28] attesting that even low levels of parasitaemia can be detected with this method in a convenient lateral flow immunoassay.

### Specificity of LF-RPA detection

In total, 77 *P. falciparum* and 11 non-Plasmodium DNAs were investigated in the LF-RPA experiments (Additional file 1: Table S1). All *P. falciparum* samples were correctly identified by a clearly visible band on the lateral flow strip (Additional file 1: Figures S1-S3). Different non-Plasmodium DNAs from prokaryotic and eukaryotic organisms were tested in cross-detection studies using an excessive amount of DNA. These tests included organisms such as *Rickettsia sibirica* which may cause symptoms similar to malaria and the closely related protozoan *Toxoplasma gondii*. In further experiments 50 ng of human DNA was used as template in the LF-RPA reaction in order to simulate the potential effect of background genomic human DNA which may be present in patient samples. The LF-RPA did not detect any of the tested negative DNA samples. The preliminary results, with a specificity of 100%, suggests that the RPA is a highly specific method and comparable to PCR or LAMP techniques [29]. The RPA primers used in this study were designed to detect *P. falciparum* only, since the initial test development focused on this species as the main cause for infection in humans. Future studies with other Plasmodium species, which are also relevant for human infections in tropical and subtropical regions, e.g. *P. vivax, P. ovale, P. malariae* and *P. knowlesi*, have to further verify the specificity of the assay. Additional tests with clinical patient samples and in-field use have to prove the effectiveness of this method as point-of-care test for the malaria parasite as well as in combination with different sample preparation methods.

### Temperature optimum

**Figure 3:**
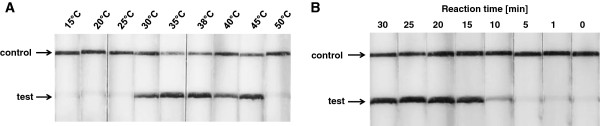
**Determination of reaction temperature and time.** The LF-RPA works effectively in a broad range of constant reaction temperatures **(A)**. After 10 min of isothermal amplification reaction, the test line is visible on the test strip. Including the incubation of 5 min the whole assay time of the LF-RPA is less than 20 min **(B)**.

The use of isothermal nucleic acid amplification methods eliminates the need for the thermocyling process necessary in the PCR. Only one constant temperature has to be applied with a temperature optimum of the RPA at 37-42°C. The LF-RPA was tested using 1 ng of gDNA in different temperature settings and found that the assay works well in a wide range of temperatures from 30-45°C, suggesting that the LF-RPA is not sensitive to gradients or small aberration in the temperature profile during the reaction (Figure 3A). The presented experiments were conducted in a Thriller-thermoincubator but the results show that the experiments could also be executed in a waterbath or, if no instrumentation is available, even by body heat. According to the supplier, the amplification reaction takes place at room temperature, albeit slower. The experiments carried out during this study did not affirm this statement since a clearly visible band on the dipstick appeared only at temperatures above 30°C. With further primer optimization and modified probes it may be possible to adapt the LF-RPA to lower temperatures. Many isothermal nucleic acid amplification techniques work at temperatures above 60°C (e.g., helicase-dependent amplification, LAMP). Using a comparatively lower temperature, the power consumption for diagnostic tests can be further reduced by the LF-RPA, which, in combination with the stability of the reaction in different temperature settings, facilitates the field use.

### Reaction time

The LF-RPA assay can be performed in less than 20 min (Figure 3B). A signal on the lateral flow dipstick can be detected after 10 min of amplification reaction at 38°C and 5 min of incubation, although the band is weaker in comparison to longer amplification times. Higher amounts of template (in the experiment shown 1 ng of genomic DNA was used) lead to a stronger signal even at shorter reaction times. An incubation period of 5 min for the lateral flow was chosen in order to assess all tests evenly. In most experiments the signal occurred faster and it may be possible to further reduce the overall assay time by an earlier read-out. In comparison to other diagnostic tests for malaria, the LF-RPA is a very rapid test, assuming that microscopy, qPCR and LAMP usually take more than one hour to complete in a laboratory setting. With its simple two-step procedure, where the first step is the reaction itself and the second step is the dilution and visualization on the lateral flow strip, the LF-RPA requires lesser hands-on time and in contrast to microscopy the procedure can be easily trained. Furthermore, the dried format of the reaction components is uncomplicated to handle and the on-site testing with an immediate read-out without further preparation steps and instrumentation eliminates sample shipment and reduces overall operator time. The use of a closed and potentially automated system, e.g., a microfluidic device, could avoid cross contamination and further simplify the handling procedure.

### Inhibitory effect

**Figure 4:**
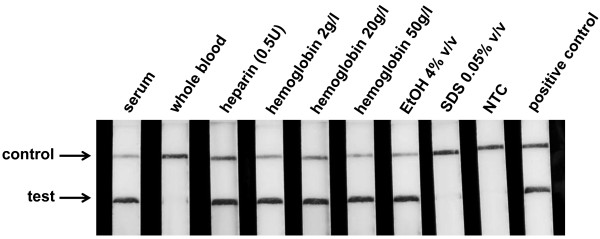
**Inhibitory experiments with lateral flow recombinase polymerase amplification.** A successful amplification and detection on the lateral flow dipstick was achieved even when high concentration of known PCR inhibitors and possible contaminants from preparation steps are present in the reaction. The LF-RPA can be carried out directly in serum samples.

Numerous substances are known to interfere with the enzymatic nucleic acid amplification. These substances can either be present in the biological sample or be introduced during preparation or the sampling process. Especially in resource-limited settings, a uniform sampling and handling might not be guaranteed. The screening for malaria is usually performed directly with blood samples or collected and stored samples are used in the form of dried blood spots for a later analysis. Blood does contain numerous potential PCR inhibitors [30], e.g., haemoglobin, lactoferrin and immunoglobulin G. Anticoagulants, used to prevent the clogging of blood samples, have an influence on the efficiency of the enzymatic amplification [31] as well as residues of substances used for sample lysis (e.g. detergents) [32], nucleic acid extraction and purification (e.g., salts, EtOH, phenol). Interference factors may hinder the enzymatic amplification reaction by direct interaction with the enzymes or by interfering with cofactors required for the enzymatic activity. In addition, a direct binding to the DNA could possibly hamper the strand synthesis by the polymerase. For the LF-RPA, several of the potential inhibitory substances that may be present in samples tested for *P. falciparum* were tested by spiking target DNA in a complex sample matrix or by adding interfering components to the reaction. In all cases a higher inhibitory concentration compared to known literature values for the standard *Taq* Polymerase were determined (Figure 4). Haemoglobin seems to have no influence on a successful LF-RPA reaction in high concentrations. No full inhibition of the reaction was observed when 50 g/l of haemoglobin or ethanol (4% v/v) was present in the assay. At template concentrations close to the limit of detection a maximum reduction of a tenfold in sensitivity was noticed (Additional file 1: Figure S4). The anticoagulant heparin (0.5 U) had not effect on the reaction while high amounts of detergent (SDS) inhibited the amplification.

The reaction was also tested by spiking 1 ng of gDNA of *P. falciparum* in human serum and human whole blood and used it for the LF-RPA. While blood serum could be directly used as matrix for a highly sensitive parasite detection (Additional file 1: Figure S4), the amplification in whole blood samples was not effective. The reason for this could be the anticoagulant sodium fluoride (NaF) which was used in this experiment or further components in whole blood which interfere in the RPA but are not yet determined. However, these preliminary results show that the LF-RPA might be more stable than common PCR systems, which would be suitable for the point-of-care setting and potentially requires only minimum clean-up and sample preparation steps. A reduced demand on preparation instrumentation would also lessen the handling time and decrease the costs for the overall diagnostic testing.

## Conclusions

Current diagnostic methods to detect *P. falciparum* suffer from a delayed time-to-result by either the reaction mechanism of the used technique or the prolonged period of time for the shipment and storage of samples to a distant, centralized laboratory. Furthermore, trained personnel are necessary to conduct the diagnostics, especially in microscopic examination but also with nucleic acid amplification-based methods where the handling of complex equipment is required. The PCR remains the usual laboratory-bound technique, although the obvious advantages of the use of nucleic acid amplification methods are the sensitivity and specific identification of the pathogen.

In this study the use of the isothermal RPA in combination with a lateral flow detection format was demonstrated. The RPA amplifies a specific target sequence at a constant reaction temperature with the support of a recombinase and a strand-displacement polymerase. The use of a special probe structure and the nuclease enzyme results in a dual-tagged DNA amplicon in a single tube reaction, which can be detected on the lateral flow strip. An assay for the detection of *P. falciparum* was established and the specificity of this LF-RPA method was determined by testing different Plasmodium strains and non-related target samples. Also the sensitivity of this method was found to be comparable to conventional PCR methods and is able to detect even low levels of parasitaemia. Additional benefits of this method are the constant low temperature necessary to conduct the amplification which, depending on the place used, might be only slightly above the surrounding temperature and the fast reaction speed from start of reaction to the analysis on the dipstick in less than 20 min. Furthermore the LF-RPA can tolerate inhibitory substances and temperature variations. Both steps of the LF-RPA, the reaction itself and the analysis on the lateral flow immunoassay, do not require any or only little instrumentation which is ideal for point-of-care testing and also results in overall lower diagnostic costs. An easily visible band on the lateral flow strip gives a clear yes/no answer that can be read by the naked eye and by untrained personnel. This is especially important in remote areas where potentially no trained healthcare worker is available. Additional tests of the LF-RPA in-field and in resource-limited settings have to prove the effectiveness of this method as a point-of-care test for the malaria parasite and further assess the reaction conditions. Especially a comparison with standard diagnostic tests and investigations with individual sample collection and preparation needs to be conducted in future studies. Also the use of a closed and potentially automated system, e.g., a microfluidic device comprising sample preparation to analyse either saliva or blood samples, is being considered to avoid cross contamination and to further simplify the handling procedure. With the PCR-like reaction scheme utilizing only two primers in the reaction, the system can be easily adapted to other DNA target sequences and also has the option to establish a multiplex reaction to potentially gain more information, e.g., subclassifications, from one sample or to establish a positive reaction control.

In summary, the LF-RPA is a practical, yet simple-to-conduct method where none or only little instrumentation is necessary. This fast and easy to read-out system could be useful as a rapid point-of-care-test to monitor malaria control efforts or to eradicate false treatment by detecting low-grade reservoirs at an early stage of the infection.

## Abbreviations

PCR: Polymerase chain reaction; RPA: Recombinase polymerase amplification; LFA: Lateral flow assay; LF-RPA: Lateral flow recombinase polymerase amplification; SSU rRNA: Small subunit ribosomal RNA; LAMP: Loop-mediated isothermal amplification; RDT: Rapid diagnostic test; NALFIA: Nucleic acid lateral flow immunoassay.

## Competing interests

The authors declare that they have no competing interests.

## Authors’ contributions

SK conceived and designed the study. VR and SK processed the samples and performed the experiments. MvNR and FFB provided the necessary infrastructure and funded the project. SK prepared the first draft of the manuscript with contribution of VR, MvNR and FFB. All authors discussed and commented on the manuscript. All authors read and approved the final manuscript.

## Supplementary Material

Additional file 1: Tested organisms_inhibitory effect. Click here for file
