# Supplementary material for: Rapid detection of Plasmodium falciparum with isothermal recombinase polymerase amplification and lateral flow analysis
Source: Malar J. 2014 Mar 15;13:99. doi: 10.1186/1475-2875-13-99 (PMC4004163; doi:10.1186/1475-2875-13-99)
Supplement: Additional file 1: — Tested organisms_inhibitory effect. [file 1475-2875-13-99-S1.pdf]

Table S1: List of tested organisms

Organism	Result in LF-RPA
P.falciparum 3D7	+
P.falciparum HB3-B2	+
P.falciparum Dd2	+
P.falciparum 3D7A	+
P.falciparum 7G8	+
P.falciparum T9/94	+
P.falciparum 7G8	+
P.falciparum HB3	+
P.falciparum W2	+
P.falciparum K1	+
P.falciparum B1SD	+
P. falciparum QC13	+
P.falciparum QC01	+
P.falciparum B4R3	+
P.falciparum 3BA6	+
P.falciparum C408	+
P.falciparum Ch3-61	+
P.falciparum 7C7	+
P.falciparum 7C16	+
P.falciparum 7C20	+
P.falciparum 7C46	+
P.falciparum 7C421	+
P.falciparum 7C424	+
P.falciparum V1/S	+
P.falciparum RO33	+
P.falciparum D10	+
P.falciparum Tm90C2A	+
P.falciparum TM90C6A	+
P.falciparum Tm90C6B	+
P.falciparum Tm91C235	+
P.falciparum 1BB5	+
P.falciparum GC06	+
P.falciparum QC34	+
P.falciparum TC08	+
P.falciparum D43	+
P.falciparum 7C140	+
P.falciparum TC05	+
P.falciparum 3BD5	+
P.falciparum SC01	+
P.falciparum SC05	+
P.falciparum Montegnard WR87	+
P.falciparum D6	+

P.falciparum 7C3	+
P.falciparum 7C12	+
P.falciparum 7C111	+
P.falciparum CH3-116	+
P.falciparum 7C126	+
P.falciparum 7C159	+
P.falciparum 7C170	+
P.falciparum FCB	+
P. falciparum FCR3CSA	+
P.falciparum ITG-2G2	+
P.falciparum ITG-2F6	+
P.falciparum Malayan Camp K-	+
P.falciparium Malyan Camp R-	+
P.falciparum Malayan Camp R+	+
P.falciparum St. Lucia	+
P.falciparum GhanaIII in Aotus	+
P.falciparum QC01	+
P.falciparum 7C424	+
P.falciparum D6	+
P.falciparum FCR3-C5	+
P.falciparum FCR-3/Gambia SL F-86	+
P.falciparum FCR9/West African	+
P.falciparum FCC-2/Hainan	+
P.falciparum FCR-3/FMG	+
P.falciparum FCR-3/Gambia Clone1	+
P.falciparum FCR-3/Gambia Clone D-3	+
P.falciparum FCR-3/Gambia Clone D-4	+
P.falciparum HB-2	+
P.falciparum T2/C6	+
P.falciparum Indo	+
P.falciparum V1/S	+
P.falciparum KMWII	+
P.falciparum MT/S1	+
P.falciparum LF4	+
P.falciparum FC27/Papua New Guinea	+
Rickettsia sibirica Strain 246	-
Toxoplasma gondii CTG ARA-SNF	-
Klebsiella pneumoniae ATCC 700721	-
Salmonella enterica DSM 14221	-
Staphylococcus aureus ATCC BAA-1720 (MRSA 252)	-
Clostridium difficile NAP7	-
Campylobacter jejuni ATCC 43431	-
E. coli K12 (Strain MG1655)	-
Yersinia enterocolitica ATCC 9610	-
Legionella pneumophila subsp pneumophila ATCC 33152	-
Human DNA derived from U937 cells	-

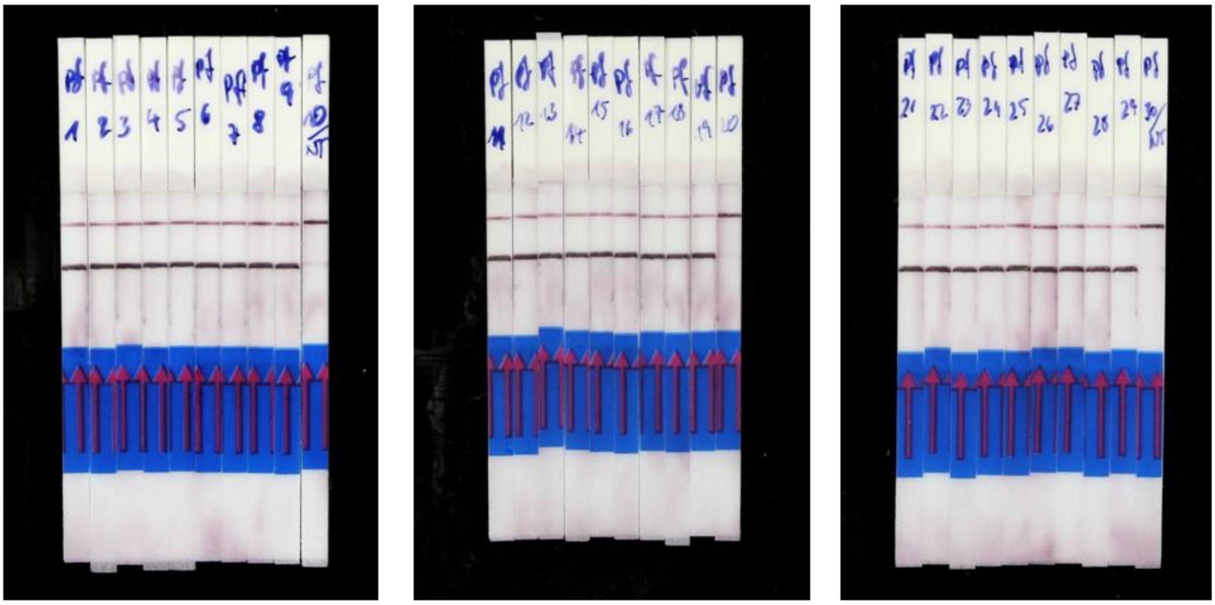

Figure S1 Left picture: *P.falciparum* TC05 (1), *P.falciparum* HB3 (2), *P.falciparum* 3D7A (3), *P.falciparum* D10 (4), *P.falciparum* RO33 (5), *P.falciparum* V1/S (6), *P.falciparum* FC27/PNG (7), *P.falciparum* T2/C6 (8), *P.falciparum* V1/S (9), NTC (10). Middle picture: *P.falciparum* 1BB5 (11), *P.falciparum* 3BD5 (12), *P.falciparum* 7G8 (13), *P.falciparum* SC05 (14), *P.falciparum* 7C12 (15), *P.falciparum* LF4 (16), *P.falciparum* KMWII (17), *P.falciparum* Indo (18), *P.falciparum* MT/S1 (19), NTC (20). Right picture: *P.falciparum* 7C7 (21), *P.falciparum* 7C159 (22), *P.falciparum* TM90C6A (23), *P.falciparum* SC01 (24), *P.falciparum* TC08 (25), *P.falciparum* D6 (26), *P.falciparum* ITG-2G2 (27), *P.falciparum* FCC-2/Hainan (28), *P.falciparum* Tm90C6B (29), NTC (30).

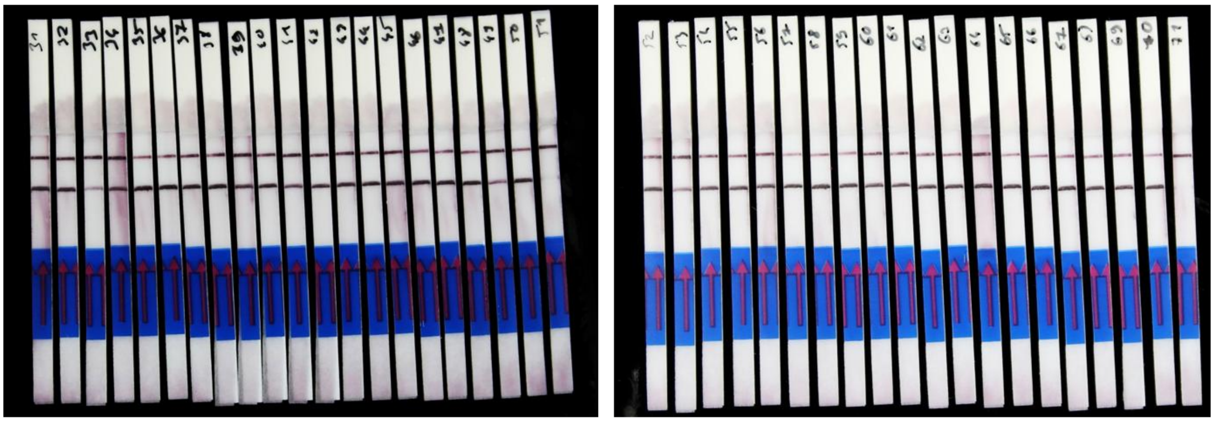

Figure S2 Left picture: *P.falciparum* Dd2 (31), *P.falciparum* CH3-116 (32), *P.falciparum* QC01 (33), *P.falciparum* 7C424 (34), *P.falciparum* FCR9/West African (35), *P.falciparum* D43 (36), *P.falciparum* 7C111 (37), *P.falciparum* D6 (38), *P.falciparum* 7C170 (39), *P.falciparum* 7C3 (40), *P.falciparum* St. Lucia (41), *P.falciparum* 7C20 (42), *P.falciparum* QC34 (43), *P.falciparum* 7C46 (44), *P.falciparum* 7C421 (45), *P.falciparum* FCR-3/FMG (46), *P.falciparum* 7C140 (47), *P.falciparum* 7C424 (48), *P.falciparum* GhanaIII in Aotus (49), *P.falciparum* FCR3-C5 (50); NTC (51). Right picture: *P.falciparum* Malayan Camp K- (52), *P.falciparum* ITG-2F6 (53), *P.falciparum* GC06 (54), *P. falciparum* FCR3CSA (55), *P.falciparum* FCR-3/Gambia Clone D-3 (56), *P.falciparum* FCR-3/Gambia SL F-86 (57), *P.falciparum* QC01 (58), *P.falciparum* Malayan Camp R+ (59), *P.falciparum* Ch3-61 (60), *P.falciparum* HB-2 (61), *P.falciparium* Malyan Camp R- (62), *P.falciparum* FCR-3/Gambia Clone1 (63), *P.falciparum* Montegnard WR87 (64), *P.falciparum* W2 (65), *P.falciparum* 7C126 (66), *P. falciparum* QC13 (67), *P.falciparum* Tm91C235 (68), *P.falciparum* C408 (69), *P.falciparum* 7C16 (70), NTC (71).

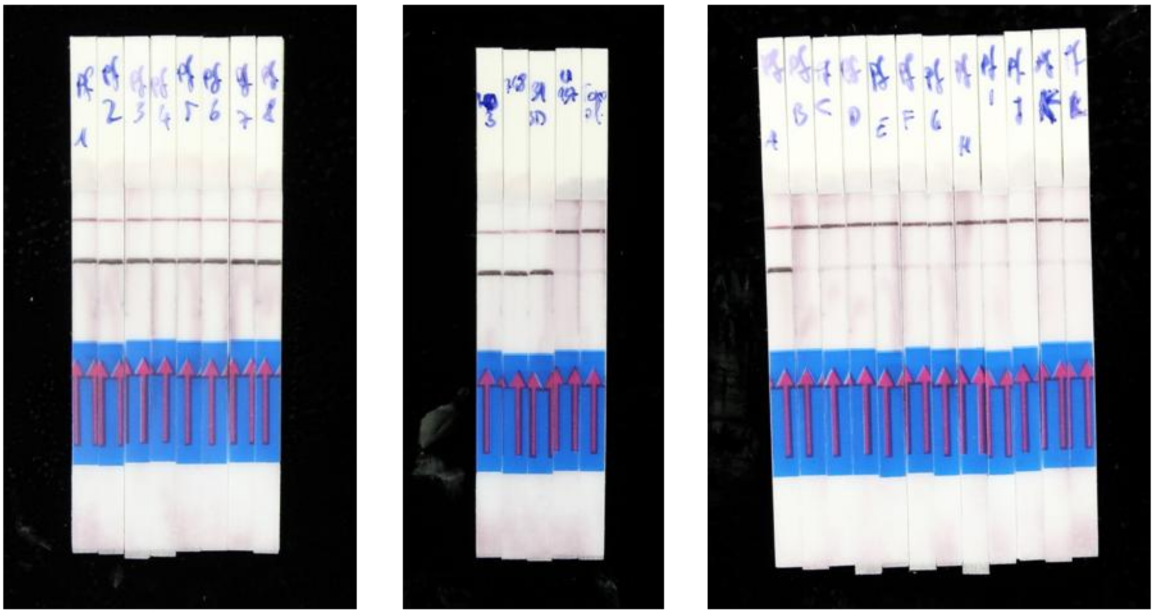

Figure S3 Left picture: *P.falciparum* 3D7 (1), *P.falciparum* FCR-3/Gambia Clone D-4 (2), *P.falciparum* FCB (3), *P.falciparum* 3BA6 (4), *P.falciparum* Tm90C2A (5), *P.falciparum* T9/94 (6), *P.falciparum* B4R3 (7), *P.falciparum* K1 (8). Middle picture: *P.falciparum* HB3-B2 (1), *P.falciparum* 7G8 (2), *P.falciparum* B1SD (3), Human DNA derived from U937 cells (4), Toxoplasma gondii CTG ARA-SNF (5). Right picture: *P.falciparum* 3D7 (A), Rickettsia sibirica Strain 246 (B), Toxoplasma gondii CTG ARA-SNF (C), Klebsiella pneumoniae ATCC 700721 (D), Salmonella enterica DSM 14221 (E), Staphylococcus aureus ATCC BAA-1720 (MRSA 252) (F), Clostridium difficile NAP7 (G), Campylobacter jejuni ATCC 43431 (H), E. coli K12 (Strain MG1655) (I), Yersinia enterocolitica ATCC 9610 (J), Legionella pneumophila subsp pneumophila ATCC 33152 (K), Human DNA derived from U937 cells (L).

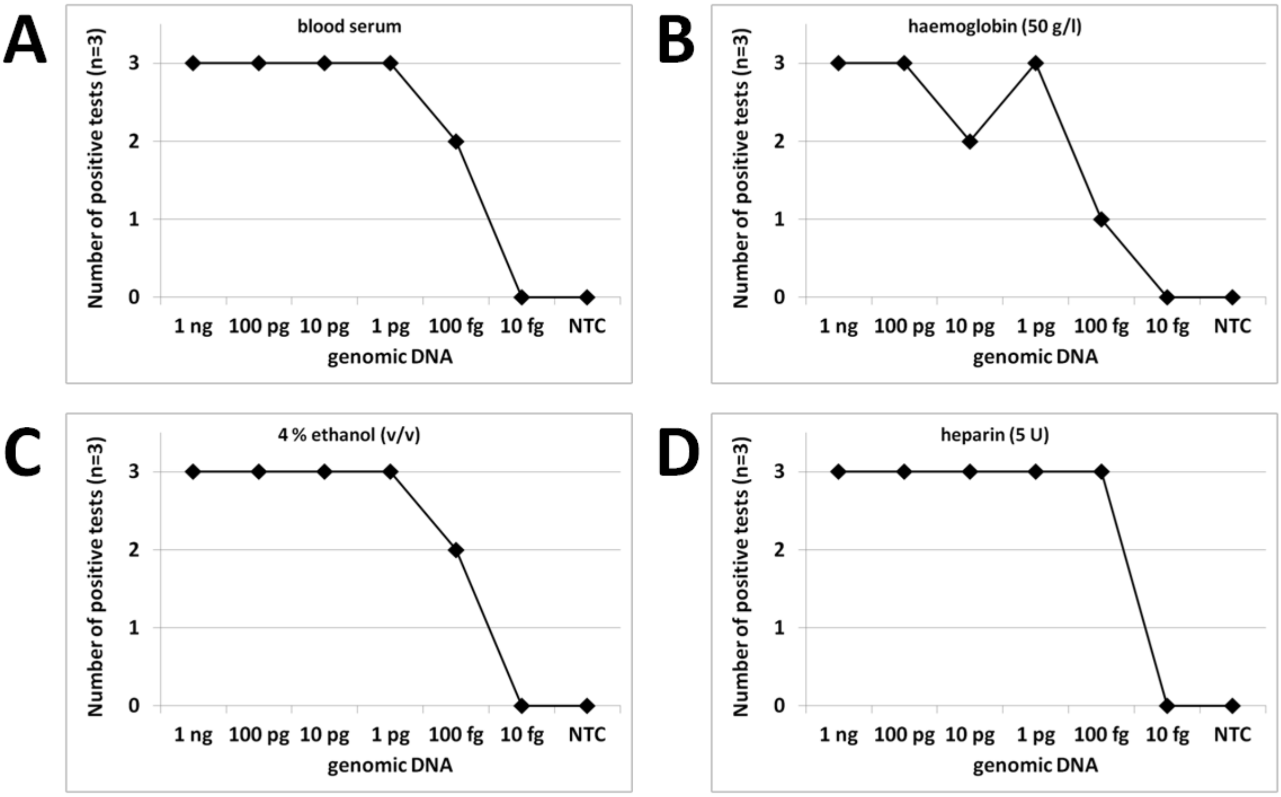

Figure S4 Efficiency of the LF-RPA in the presence of different known PCR inhibitors using a dilution series of *P. falciparum* 3D7 genomic DNA in triplicate experiments. The LF-RPA was performed by spiking different amounts of Plasmodium - DNA in human serum samples **(A).** In further experiments 50 g/l haemoglobin **(B)**, 4 % ethanol (v/v) **(C)** and 5 U heparin **(D)** were added to the reactions with different amounts of template DNA. The inhibitory substances do not significantly alter the sensitivity of the LF-RPA even if applied in relatively high concentrations. Heparin does not affect the amplification efficiency at all, while the other substances and especially haemoglobin only have an influence on the reaction at template concentration close to the limit of detection (100 fg).
